# Identification of Catecholamine and Drug Target α_2A_-Adrenoceptor in Human Testis and Human Testicular Peritubular Cells

**DOI:** 10.3390/jcm13154357

**Published:** 2024-07-25

**Authors:** Welter Harald, Kreitmair Nicole, Schneider Michaela, Herrmann Carola, Schmid Nina, Stepanov Youli, Fröhlich Thomas, Köhn Frank-Michael, Pickl Ulrich, Trottmann Matthias, Mayerhofer Artur

**Affiliations:** 1 Biomedical Center, Cell Biology, Anatomy III, Faculty of Medicine, Ludwig Maximilian University of Munich, 82152 Planegg-Martinsried, Germany; welter@bmc.med.lmu.de (W.H.); nicole.kreitmair@bmc.med.lmu.de (K.N.); michaela.schneider@bmc.med.lmu.de (S.M.); carola.herrmann@bmc.med.lmu.de (H.C.); nina.schmid@bmc.med.lmu.de (S.N.); 2 Gene Center Munich, Laboratory for Functional Genome Analysis (LAFUGA), Ludwig Maximilian University of Munich, 81377 Munich, Germany; stepanov@genzentrum.lmu.de (S.Y.);; 3 Andrologicum, 80331 Munich, Germany; info@andrologicum.com; 4 Urologie und Andrologie am Promenadenplatz, 80333 Munich, Germany; u.pickl@web.de (P.U.); m.trottmann@gmx.de (T.M.)

**Keywords:** alpha-adrenoreceptor, testis, contraction, peritubular cell, human male fertility, human testis, cytokines, inflammation, clonidine, sperm transport

## Abstract

**Background:** Clonidine has been used in clinical medicine, e.g., to treat high blood pressure and other conditions. Animal studies have linked its use to impairments of male reproductive functions, and although only a few reports exist for the human species, such actions may exist in man as well. The underlying reasons and, specifically, possible actions of clonidine at the level of the testis are not known. **Introduction:** Clonidine is an agonist at the α_2A_-adrenoceptor (ADRA2A), which, as data bank mining indicated, is expressed by several cells of the human testis. The human testis and most of its cells are, however, not readily accessible to experimental testing. Cells from the peritubular wall compartment (human testicular peritubular cells; HTPCs) are the exception. **Methods and Results**: As shown by immunohistochemical/immunocytochemical and PCR techniques these cells express ADRA2A and retain expression upon isolation and culture. When tested over a concentration range (1–1000 µM) and 24 h, clonidine did not visibly affect HTPC morphology but significantly stimulated *IL6* mRNA levels in a concentration-dependent manner. ELISA measurements of cell culture supernatants confirmed a stimulatory action of clonidine (10 µM) on secreted IL6. When examined in collagen gel contraction assays of HTPCs, clonidine (10 µM) exerted a slight relaxing action, while a proteomic study revealed that clonidine (10 µM) did not significantly change cellular protein abundance of HTPCs after 24 h (data available via ProteomeXchange with identifier PXD052220). **Conclusion:** Thus, ADRA2A-bearing cells in the human testis are targets for catecholamines and drugs such as clonidine. The results of this HTPCs-focused study only show the tip of the iceberg. It is likely that catecholamines/catecholaminergic drugs have the potential to interfere with human testicular functions.

## 1. Introduction

Clonidine is in clinical use to treat high blood pressure and attention-deficit/hyperactivity disorder (ADHD, monotherapy, or adjunctive therapy) and other conditions [1,2,3,4]. Clonidine is an agonist at the α_2A_-adrenoceptor (ADRA2A; [5,6]), and its modes of action in the treatment of high blood pressure involve relaxing actions on vascular smooth muscles [7,8]. This is thought to be an indirect action, namely the consequence of a central inhibition of the sympathetic system.

Of note, the use of clonidine was implicated in male fertility problems; however, only limited information is available [9]. In man, the reported adverse effects are erectile dysfunction [10]. Yet, when clonidine actions were studied in rats, the animals became either unable to ejaculate or were sterile [11]. Furthermore, sperm transit time through the epididymis was significantly shorter in clonidine-treated rats than in controls. This is somehow in contrast to the reported relaxing ADRA2A-mediated actions in the epididymis and the testicular capsule [12,13], which were also seen in rats. Both of these tissues contain smooth muscle cells, which may thus be targeted. However, the multiple adverse effects of clonidine on several male reproductive organs and their functions, specifically the testis, remain to be further explored.

Clonidine, as mentioned, is an agonist at the catecholamine receptor ADRA2A. To explore whether it is expressed in the human testis, we initially searched for testicular catecholamine receptors [14] in the Human Protein Atlas (https://www.proteinatlas.org, accessed on 27 September 2023). Results revealed the expression of all catecholamine-receptors, except *ADRB3*, in the human testis and indicated expression by various cells. With respect to testicular peritubular cells, which build the peritubular wall compartment in man, results pinpointed the expression of *ADRA1B*, *ADRA2A*, *ADRA2B*, and *ADRA2C*.

Human testicular peritubular cells (HTPCs) [15,16] can be isolated and examined in vitro. They are a highly adequate cellular model for this human testicular compartment, as indicated by a recent single-cell RNA sequencing study [17] because they retain the great majority of their in situ characteristics. We have previously, by employing RT-PCR, identified *ADRA1B* in HTPCs [18]. Therefore, the peritubular expression of *ADRA1B* was to be expected (see https://www.proteinatlas.org/ENSG00000170214-ADRA1B/single+cell+type/testis; accessed on 27 September 2023) and confirmed our previous results. The mentioned data mining, however, also identified *ADRA2A* in peritubular cells, next to Leydig cells, spermatocytes, spermatids, Sertoli cells, and endothelial cells (https://www.proteinatlas.org/ENSG00000150594-ADRA2A/single+cell+type/testis; accessed on 27 September 2023).

Our previous studies in HTPCs [19] revealed that the natural catecholamine, epinephrine, which interacts with all catecholamine receptors, including ADRA1B and ADRA2A, stimulated COX-2, MCP1, and IL6, implying that this natural catecholamine may promote a pro-inflammatory milieu in the testis, e.g., under conditions of chronic stress. Similar results were found when phenylephrine was studied, which primarily acts on ADRA1B [19].

The paucity of information on catecholamine actions in the human testis, in particular the lack of any information on the potential actions of clonidine [20,21] and the use of clonidine in men and boys, led us to address the question of whether clonidine via ADRA2A may also be able to regulate the functions of HTPCs.

## 2. Materials and Methods

### 2.1. Human Samples, Cell Culture, Reagents and Treatments

Testicular tissue of patients (29–55 years who were undergoing testicular sperm extraction) was used for the isolation of HTPCs and for immunohistochemical experiments as described earlier [22]. The study was conducted according to the guidelines of the Declaration of Helsinki. Written informed consent from all patients was obtained and the local Ethics Committee (Technical University of Munich, Faculty of Medicine; project 491/18S-KK; date of approval 14 November 2018) approved the study. In total, HTPCs from nine different donors were included in this study. In detail, for RT-PCR and dose–response experiments, identical donors were used (*n* = 3/experiment), whereas two additional patients were included for qPCR and proteomic analyses (*n* = 5/experiment). Immunocytochemistry studies were performed with HTPCs of four donors (three of which were also used in qPCR studies), while cell contractility assays were conducted with three further patient-derived cells not used in the aforementioned experiments. Cell culture experiments were conducted with freshly isolated or cryopreserved HTPCs (passages 7–12). They were seeded at a density of 2.5 × 10^5^ cells per 60 mm dish—and cultured in Dulbecco’s Modified Eagle Medium high glucose (DMEM; Gibco, Paisley, UK) with 10% (*v*/*v*) fetal calf serum (FCS; Capricorn Scientific, Ebsdorfergrund, Germany), and 1% (*v*/*v*) penicillin/streptomycin (P/S; Gibco, Paisley, UK) at 37 °C, 5% CO_2_ and 95% humidity for 24 h [15,23]. For the experiments, cells were serum-starved for 24 h and then stimulated in serum-free medium with clonidine (2-[(2,6-Dichlorophenyl) amino-2-imidazoline hydrochloride; #0690 Tocris Bioscience™, Wiesbaden-Nordenstadt, Germany). It was dissolved in PBS, and therefore, PBS served as solvent control. Increasing concentrations of clonidine (1 µM, 10 µM, 100 µM and 1000 µM for up to 24 h) were tested, and morphology and *IL6* levels were monitored. A concentration of 10 µM was finally chosen as effective and used in most of the experiments. This concentration was used in other reports studying contractility of the epididymis or capsule of the rat testis as well [13,24].

### 2.2. Isolation of RNA, Reverse Transcription (RT-PCR), and Quantitative Real-Time PCR (qPCR)

To perform qPCR analyses, total RNA from cultured HTPCs of five individual donors was extracted using QIAGEN RNeasy Mini-Kit (QIAGEN, Hilden, Germany) according to the manufacturer’s instructions. 1000 ng RNA were reverse transcribed using SuperScriptTM II (Invitrogen, Carlsbad, CA, USA). Final dilutions corresponding to 2 ng per qPCR-reaction were prepared and subjected to quantitative RT-PCR reactions using QuantiFast SYBR Green PCR Kit (Qiagen, Hilden, Germany) on the LightCycler 96^®^ System (Roche Diagnostics, Penzberg, Germany) as described [22]. Primers were designed and selected using the online tool primer3 (http://primer3.wi.mit.edu, accessed on 15 September 2023; primers are also listed in Table S1). Samples were run in duplicate and cq-values were picked to process data via the 2^−ΔΔCq^ calculation method [25].

### 2.3. Immunohisto- and Immunocytochemistry

Testicular tissue from patients with normal spermatogenesis and mixed atrophy was fixed in Bouin’s solution and embedded in paraffin, as mentioned before, including citrate buffer pH 6.0 for antigen retrieval [26]. An anti-ADRA2A primary antibody (#ab85570; diluted 1:100–1:200; affinity-purified, polyclonal rabbit anti-human ADRA2A, Abcam, Berlin, Germany) was used. Negative controls consisted of omission of the first antibody, rabbit normal serum, or incubation with rabbit IgG isotype. After counterstaining with hematoxylin, sections were analyzed using a Zeiss Axioplan microscope (Carl Zeiss Microscopy, Oberkochen, Germany), and staining was documented with a Progres Gryphax^®^ microscopy camera and software (Jenoptik, Jena, Germany). For immunocytochemistry, HTPCs from four different donors were seeded overnight onto round 12-mm glass coverslips (20,000 cells/coverslip). Thereafter, cells were fixed with buffered 4% formaldehyde solution, pH 6.9 (Merck, Darmstadt, Germany) for 10 min followed by three washing steps with PBS, and incubation in blocking buffer (PBS supplemented with 5% normal goat serum (Sigma-Aldrich, Merck, Darmstadt, Germany) and 0.1% Triton X-100) for 30 min at RT. Subsequently, samples were incubated overnight at 4 °C with the same primary antibody (diluted 1:50) as mentioned above. The secondary antibody, goat anti-rabbit Alexa Fluor 555 (#21428; 1:1000, Thermo Fisher Scientific, Waltham, MA, USA), diluted in blocking buffer for 1 h at RT, was used to visualize ADRA2A protein and nuclei were counterstained with DAPI (1 µg/mL, diluted in H_2_O; Sigma-Aldrich, Merck, Darmstadt, Germany). Finally, samples were washed in PBS and water before embedding in Dako mounting medium (Agilent Technologies, Santa Clara, CA, USA). Images were acquired using a Zeiss Z1 Axio Observer microscope equipped with a 40×/63× PlanApo oil immersion objective (Carl Zeiss Microscopy, Jena, Germany) and ZEN acquisition software (ZEN blue 2.6).

### 2.4. IL6 ELISA Measurements

We used the commercial IL6 Human ELISA Kit (# KHC0061, Thermo Fisher Scientific, Waltham, MA, USA) according to the manufacturer’s instructions. Culture supernatants of clonidine-treated (10 µmol/L) and untreated (DMEM + PBS) control cells derived from 5 donors were harvested after 24 h. Samples were run in duplicates, and values were normalized to cellular protein.

### 2.5. Cell Contractility Assay

Collagen I-3D Gelling Kit (#8178, Sciencell, Carlsbad, CA, USA) was used for contractility experiments. In brief, 24-well plates were coated with 0.2% BSA (diluted in PBS) and incubated for 4 h at 37 °C. Remaining BSA was withdrawn, and plates were allowed to dry for further 60 min at RT. Collagen matrices containing 100,000 cells in 500 µL volume per well were seeded into a 24-well plate. After 1 h of polymerization at 37 °C, collagen gels were covered with serum-free medium and cultured with daily changes of medium for 72 h. Clonidine (10 µM), solvent control (PBS), and 30% FCS (positive control) were added in duplicates, and contraction of the free-floating collagen gels was monitored over 24 h. Images of gel matrices 0 h (before treatment), 3, and 24 h (after treatment) were taken and analyzed using Fiji [27]. The contractility of the examined HTPCs (derived from 3 individual donors) was finally tested by adding 30% FCS for 1 h after the 24-h experiment. Changes in gel size after clonidine treatment were compared to 0 h. To evaluate cell viability at the end of the experiments, HTPCs were incubated with Calcein-AM (Thermo Fisher Scientific, Waltham, MA, USA), diluted 1:1000 in DMEM for 15 min at 5% CO_2_, 37 °C and 95% humidity. Subsequently, fluorescence was detected using a Leica microscopy system (DM IL LED microscope, Leica DFC3000G microscopy camera, and the included software Leica Application Suite X, version 3.7.0.20979, Leica Microsystems; GmbH, Wetzlar, Germany).

### 2.6. Nano LC-MS/MS-Based Proteomic Analysis

Samples of HTPCs from 5 different donors exposed for 24 h to clonidine (10 µM) and respective controls were cryopulverized and processed as described [28]. Protein digestion was performed in two steps: (i) Lys C (Fujifilm Wako, Neuss, Germany) 1:100 enzyme/protein ratio for 4 h at 37 °C; (ii) dilution of samples with 50 mmol/L NH_4_HCO_3_ to 1 mol/L urea (final conc.), addition of porcine trypsin (Promega, Madison, WI, USA), 1:50 enzyme/protein ratio, digestion overnight at 37 °C. Mass spectrometry analysis was performed similarly as already described for Dex-treated HTPCs [29]. In brief, an Ultimate 3000 RSLC nano-chromatography system connected to a Q Exactive HF-X mass spectrometer (Thermo Scientific, Waltham, MA, USA) was used. Mobile phase A was 0.1% formic acid in water, and as mobile phase B, 0.1% formic acid in acetonitrile was used. Peptides were separated at a flow rate of 250 nL/min on an EASY-spray column (Pepmap™ RSLC C18, 2 µm, 75 µm × 50 cm, Thermo Scientific, Waltham, MA, USA) with a two-step gradient from 3% B to 25% B in 30 min followed by a ramp to 40% B for 5 min. Spectra were acquired with a top 15 data dependent method and analyzed using MaxQuant (V1.6.11.0). As a sequence database, the Homo sapiens subset of the SwissProt database (retrieved in March 2022) was used. The heatmap and volcano plots were generated in Perseus (V1.6.5.0) [30]. The mass spectrometry proteomics data have been deposited to the ProteomeXchange Consortium (http://proteomecentral.proteomexchange.org) via the PRIDE partner repository [31] with the dataset identifier PXD052220.

### 2.7. Statistical Analysis

Statistical analyses of qPCR and ELISA data were performed using GraphPad Prism 6.0 Software (GraphPad Software Inc., San Diego, CA, USA). qPCR data were analyzed via *t*-test of 2^−ΔΔCq^ values, as were IL6 levels in the supernatant via one-sample *t*-tests of non-normalized values. Paired *t*-test was used to compare two groups. A probability value of *p* < 0.05 was considered significant.

## 3. Results

### 3.1. ADRA2A Expression in Human Testicular Biopsies of Adult Men

**Figure 1:**
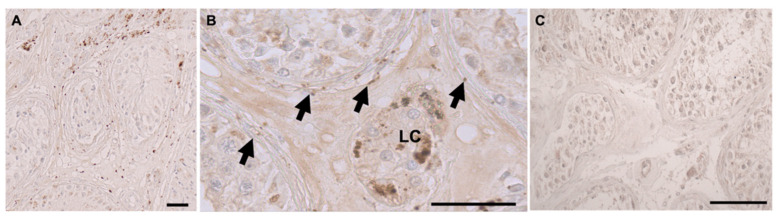
Immunohistochemistry of ADRA2A in the human testis. (**A**,**B**) In representative human testicular samples of two mixed atrophy patients, ADRA2A staining is found in peritubular, myoid cells (arrows in (**B**)) building the wall of seminiferous tubules and in interstitial cells, presumably Leydig cells (LC in (**B**)). Nuclei were slightly stained with hematoxylin. (**C**) When the first antibody was substituted by an IgG isotype antibody, a slight and faint overall brownish staining was observed in an additional third donor sample. Scale bars 50 µm.

In agreement with expression data from the HPA, ADRA2A-immunoreactive cells were found in cells of the tubular compartment of human testes sections, Leydig cells, and also in some peritubular cells (Figure 1). In these, punctate signals were observed. Signals were found in peritubular cells of tubules with normal and impaired spermatogenesis but were absent in all controls, including the IgG control slides.

### 3.2. ADRA2A Expression in Cultured Human Testicular Peritubular Cells (HTPCs)

**Figure 2:**
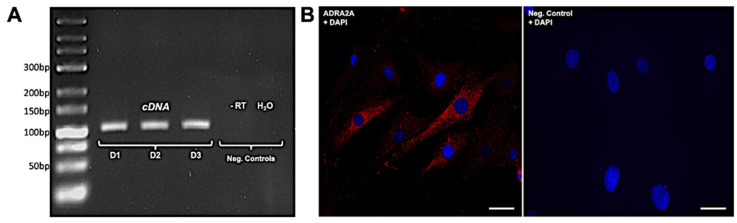
Expression of ARDRA2A in human testicular peritubular cells (HTPCs). (**A**) Representative RT-PCR products of HTPCs derived from three different donors (D1–3) show a single band, which, upon sequencing, was found to correspond to human *ADRA2A*. Negative controls, including minus RT (-RT) and H_2_O instead of cDNA, exhibit no band. (**B**) Immunofluorescence of ADRA2A protein (red) in cultured HTPCs. Note the heterogenous signal intensity of ADRA2A in individual cells. Omission of the first antibody (Neg. Control) revealed no staining. Nuclear DNA (blue) was stained with DAPI. Scale bars 40 µm.

Upon isolation and culture of HTPCs derived from three individual donors, *ADRA2A* mRNA expression was retained, as shown by RT-PCR (Figure 2A). Using immunocytochemistry, dense, fine, and dispersed ADRA2A-immunoreactive signals were observed in the cytoplasm of some HTPCs, while in others, immunoreaction was absent or weak (Figure 2B). This heterogeneity was observed in all HTPCs and is in line with data extracted from scRNAseq (Figure S1, extracted from [17]). Nevertheless, the expression of ADRA2A in HTPCs was profound and detectable in 60–70% of cells derived from all four individual patients tested. This led us to explore the actions of clonidine.

### 3.3. Dose Response of Clonidine on Cell Viability and IL6 mRNA Expression of HTPCs

Results of a dose–response experiment with increasing concentrations of clonidine ranging from 1 µM, 10 µM, and 100 µM to 1000 µM for 24 h did not indicate obvious toxic actions of clonidine (Figure S2).

**Figure 3:**
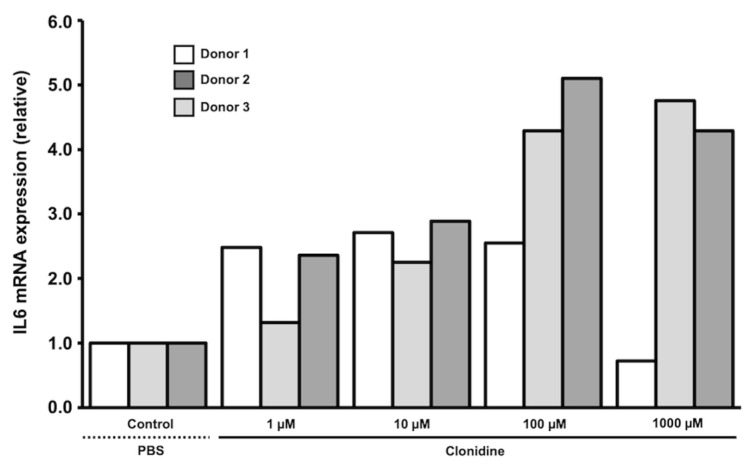
Regulation of *IL6* mRNA levels by increasing concentrations of clonidine in cultured HTPCs. Clonidine treatment (1 µM, 10 μM, 100 μM, and 1000 µM) for 24 h provoked a concentration-dependent increase in *IL6 mRNA* in two out of the three donors. qPCR data were normalized to *GAPDH* and expressed relative to control (PBS); *n* = 3 individual donors.

A dose–response experiment with the same HTPCs as used in Section 3.2 was also performed and *IL6* transcript levels were determined. In two out of the three donors, we found that clonidine, in a concentration-dependent manner, increased the mRNA levels of *IL6* (Figure 3). However, in cells of the third donor, the highest concentration of clonidine rather decreased *IL6*. We chose to study clonidine at 10 µM, which was used in previous studies [13,24] and produced a robust > 2-fold response.

### 3.4. Effect of 10 µM Clonidine on Typical HTPCs Transcripts and IL6 Secretion

**Figure 4:**
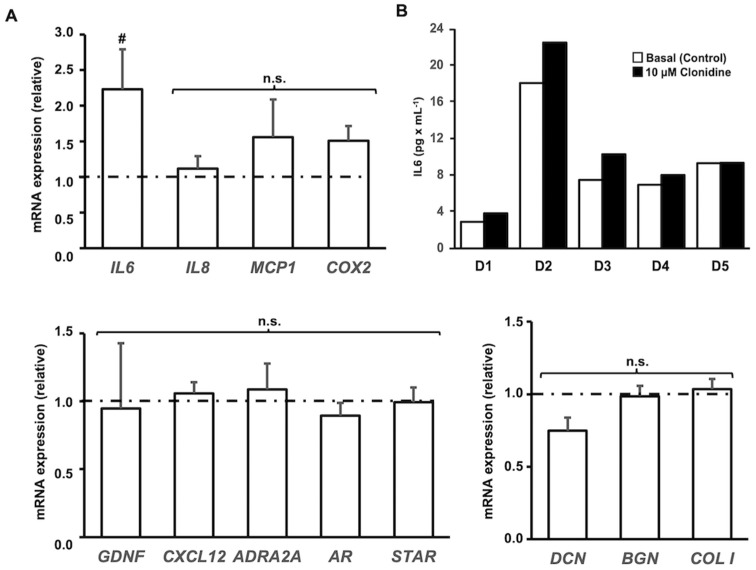
The addition of 10 µM clonidine to cultured HTPCs increased *IL6* mRNA abundance but did not affect mRNA levels of other typical HTPC genes. (**A**) Cultured HTPCs stimulated with clonidine (10 µM) respond with a significant increase (*p* < 0.05) in *IL6* mRNA but not in *IL8*, *MCP1*, or *COX2* levels after 24 h compared to control (dashed line). Similarly, transcript levels of *GDNF*, *CXCL12*, *ADRA2A*, *AR*, *STAR*, as well as the levels of the extracellular matrix components *DCN*, *BGN*, and *COLI* do not significantly change (*p* > 0.05) after application of clonidine. Bars are mean + SEM after 24 h; *n* = 5 donors, normalized to control conditions (dashed line). # denotes statistical significance, # *p* < 0.05; n.s. not significant. (**B**) Results of ELISA measurements of secreted IL6 from 5 individual donors (D1−5) in response to stimulation with clonidine (10 µM for 24 h) are shown. Note robust increases but strong inter-individual differences in basal levels and in the degree of the increments.

Employing 10 µM of clonidine, we also examined levels of typical HTPC transcripts, namely *GDNF*, *CXCL12*, *IL8*, *MCP1*, *COX2*, *AR*, *StAR*, *DCN*, *BGN*, *COLI*, and *ADRA2A*, using cells from five patients. Results revealed that only *IL6* levels were significantly and more than twofold increased, while all others were unaffected by the 10 µM clonidine treatment for 24 h (Figure 4). An IL6 ELISA experiment (10 µM clonidine; 24 h) revealed slight (3%) to robust (up to 38%) increases in 5 individual donors, but again, strong inter-individual differences even in basal levels of IL6 secretion became apparent (Figure 4B).

### 3.5. Relaxing Actions of 10 µM Clonidine on HTPCs

**Figure 5:**
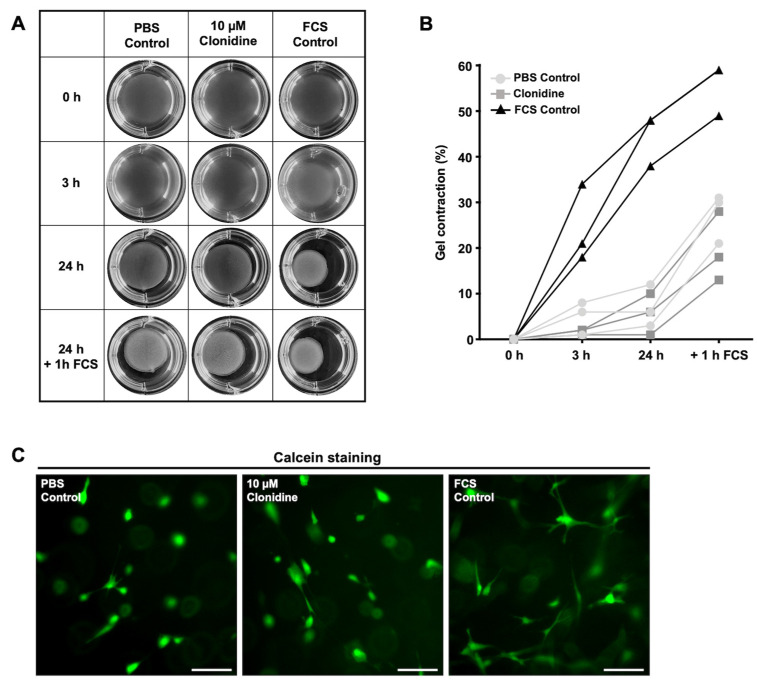
Clonidine has a slight, relaxing action in HTPCs. (**A**,**B**) Clonidine (10 µM), solvent control (PBS Control), and 30% FCS (FCS Control, positive control) were added, and contraction of the free-floating collagen gels was monitored over 24 h. Images of gel matrices 0 h (before treatment), 3 h, and 24 h (after treatment) are depicted. Gel contraction assay indicates slight relaxing actions of clonidine after 3 and 24 h on basal contractility (basal contraction and reduction of the gel size by 5% vs. 1.6%, respectively, after clonidine treatment). (**C**) The viability of HTPCs at the end of the experiment is confirmed by green fluorescence (Calcein-AM uptake). Scale bars 100 µm.

Gel contraction assays were next performed (10 µM clonidine; *n* = 3). A slight relaxing action of clonidine after 3 h and 24 h (Figure 5A,B) became visible. While the mean reduction of the gel size was 5% (range 1–8%) in the control group, it was 1.6% (range 1–2%) in the clonidine treatment group. For clarity, the percentage of gel contraction is also shown in Figure S3. When, after the 24 h period, FCS, which induced HTPCS to contract, was added, gel size and, thus, the overall contractile state of clonidine-treated samples remained lower than in the controls. The mean of gel contraction of the control was 27.7% (range 21–31%), while the one of the clonidine-treated cells was 19.6% (range 13–18%). The viability of the examined HTPCs was unchanged, as assessed by Calcein-AM uptake at the end of the experiments (Figure 5C).

### 3.6. No Significant Alterations of the Cellular Proteome in HTPCs after Clonidine Exposure

A total of 1746 proteins were identified, and 902 proteins were quantified for the herein described results. The proteomic analysis of samples (*n* = 5 donors) from clonidine-treated HTPCs (10 µM; 24 h) was carried out using the Welch’s *t*-test (two-sample test, with truncation using permutation-based FDR of 0.05) with significance cut-off criteria of *p*-value ≤ 0.05 and Log_2_-Fold Change ≥ 0.6, i.e., an increase or decrease in protein abundance by 50%. This analysis indicated no changes in cellular protein abundance. High experimental homogeneity was demonstrated by the Pearson correlation analysis for the homogeneity of biological replicates, thus supporting the lack of proteomic variance between control and treated samples. The observations are summarized in Figure S4.

## 4. Discussion

Clonidine acts via ADRA2A. Expression of this catecholamine receptor by several cells of the human testis, namely Leydig cells, spermatocytes, spermatids, Sertoli cells, endothelial cells, and peritubular cells, became evident when we searched the HPA single-cell expression database. Immunohistochemistry performed in paraffin-embedded testes samples confirmed this widespread expression, in general. Yet, immunostaining in the tubular compartments was weak overall; staining of peritubular cells was not uniform while staining in Leydig cells was strongest. Based on these results, one must assume that these cells are targets for clonidine, as well as for other related drugs and natural catecholamines. Most of them can, however, not be readily examined. HTPCs are the noteworthy exception and were studied.

HTPCs build the wall of the seminiferous tubules in the human testis and form several cellular layers, yet immunoreactive ADRA2A was detected in only some HTPCs in testicular sections. A comparable situation was reported for the wall of the human bladder [32], where few ADRA2A-positive interstitial cells were previously identified between the different smooth muscle cell layers. This appears to be human-specific, as they were not found in mouse bladders. The ADRA2A-positive cells were suggested to be involved in nerve conductivity and, thus, the regulation of bladder contractility. In analogy, the peritubular wall of the human testis is innervated, and hence, such a role for ADRA2A-positive cells may also be conceivable.

HTPCs are testis-specific smooth muscle cells [17], which have the ability to contract and relax, and in vivo, they play an important role in intratesticular sperm transport [33]. The contractile abilities of HPTCs were therefore examined in collagen gel contraction assays, and our results revealed that clonidine exerted weak relaxing actions.

As clonidine is used to treat high blood pressure, its modes of action involve relaxing actions on vascular smooth muscles [7,8]. However, this is thought to be an indirect action and represents the consequence of a central inhibition of the sympathetic system by clonidine. This puts the coupling of the vascular smooth muscle with its innervating sympathetic nerve fiber in the center.

Lacking such innervation, the underlying mechanism for the relaxing action of clonidine in isolated HTPCs grown in collagen gels must be different and direct ones. It may be possible that clonidine inhibits spontaneous transient increases in Ca^2+^ and thereby its intracellular signal transduction, actions which were reported in tracheal smooth muscle [34].

Relaxing ADRA2A-mediated actions were also reported in experiments with rat epididymis and the testicular capsule [12,13,24] at concentrations of 10 µM, which corresponds to the one used in our experiments. Of note, more recent data indicated that clonidine (100 nM), albeit in a somewhat other experimental setting, can also induce phasic contractions of the rat epididymis [35]. The mechanisms for clonidine actions to affect the contractile abilities of smooth muscle cells may thus involve direct and indirect ones. They remain to be further studied.

Next to a role in sperm transport, HTPCs have further functions. Among others, they produce ECM-proteins, cytokines, prostaglandins, and growth factors [16,36,37]. They express AR and StAR [38,39]. Therefore, we explored whether clonidine (10 µM) may affect these properties by using a sensitive qPCR screen.

No significant changes in the levels of *ADRA2*, *AR*, *StAR*, *GDNF*, CXCL12, or ECM components (*DCN*, *BGN*, *COL1*) were found. Yet, *COX2*, *IL8*, and *MCP1* were somewhat, yet not significantly, increased upon the addition of clonidine at 10 µM for 24 h. *IL6* levels were significantly elevated by 10 µM (and even more by higher concentrations), and therefore, we examined secreted IL6 levels in samples from additional five patient-derived cells. Like in earlier studies (e.g., [26]), a strong heterogeneity became apparent, which is most likely due to the fact that the HTPCs stem from individual patients. Yet, in all sample pairs, IL6 levels increased after the addition of clonidine. Of note, IL6 was also elevated by epinephrine in a previous study [18], i.e., the natural ligand of ADRA2.

IL6 is a major circulating cytokine in the human species. IL6 is of interest because a number of actions in the testis were described [40] with respect to spermatogenesis and Leydig cell functionality. Most studies were, however, performed with rodents, including IL6-mutant mice. For example, a study by Morales-Montor et al. [41] showed a role in promoting the aromatization of testosterone. This result is in line with data from rat testicular organotypic incubations, in which clonidine increased estradiol production [42]. Wang et al. [43] further showed that in rodent testis, IL6 also inhibits Leydig cell differentiation. The results of a study in human volunteers revealed that injections of IL6 reduced testosterone production in these men [44], most likely by acting at the levels of the testis. Provided that the stimulating actions of clonidine on IL6 release by HTPCs in vitro exist in vivo, negative consequences on (human) testicular functions might, therefore, be expected as a consequence of long-term clonidine use in man.

The mass spectrometry analysis of HTPC proteomes did not reveal any significant proteomic alterations after 24 h clonidine treatment. A likely reason for this is that proteins with low abundance, such as cytokines or growth factors, are often not detected with this approach. To detect those, other focused approaches were required until now. However, with technological advances in mass spectrometry, such changes may become detectable in the future. Another reason for this result may be the fact that ADRA2A is known to rapidly desensitize [24,45,46]. The constantly available clonidine in the medium of HTPCs in our experiments may, therefore, have caused cells to become unresponsive, and hence, consequences at the protein levels were not measured. The observed increased IL6 release upon clonidine was measured by ELISA and PCR and may furthermore be a rapid and initial consequence.

Clearly, beyond the just mentioned methodological limitation, our study in human testicular cells has a number of other limitations. It was focused on the only available human testicular cell type, which expresses ADRA2A, i.e., peritubular cells and clonidine. Clonidine actions on human Leydig cells and other immunoreactive cells could not be studied. Hence, we only obtained a partial and cell-specific answer to the question of how clonidine may affect the human testis. Regarding future experimental studies in HTPCs, they also should include different short-term stimulation, different time points, and concentrations.

Is this question of the potential actions of clonidine on the human testis really of clinical relevance? The use of clonidine in ADHD and the treatment of hypertension is certainly limited, as new data indicate [47,48]. Yet, as paternal age is increasing in developed countries, so is hypertension in men, and thus the use of anti-hypertensive drugs, including clonidine [49,50] (https://www.cdc.gov/nchs/products/databriefs/db364.htm; accessed on 2 March 2024). Thus, the presently limited clinical relevance may become more important in the future.

Also, other drugs, which act via ADRA2A, would be of interest. Dexmedetomidine is among these agonists and is also used in clinical practice, e.g., for its sedative properties. A further agonist is the “Zombie drug” xylazine [51] (https://www.guidetopharmacology.org/GRAC/LigandDisplayForward?ligandId=523; accessed on 2 March 2024). Xylazine is used in veterinary medicine as a large animal sedative. It has anesthetic, muscle relaxant, and analgesic effects. In recent years, it became known because it has been added to other illicitly-manufactured drugs and has severe side effects [51,52]. Based on our study, we conclude that they might include as yet unknown actions at the level of the testis.

## 5. Conclusions

Our study is limited to clonidine and its actions in HTPCs. We identified an influence on contractility and IL6 production. The results obtained in our study are however most likely only the tip of the iceberg. Next to ADRA2A, several other catecholamine receptors exist in the human testis and the receptor-bearing cells represent targets for natural catecholamines and drugs, which, therefore, most likely affect the male gonad and its functions.

## Data Availability

The mass spectrometry proteomics data have been deposited to the ProteomeXchange Consortium (http://proteomecentral.proteomexchange.org) via the PRIDE partner repository with the dataset identifier PXD052220.

## Supplementary Materials

The following supporting information can be downloaded at: https://www.mdpi.com/article/10.3390/jcm13154357/s1, Table S1: Information of PCR-Primer (AT: Annealing Temperature); Figure S1: UMAP plot of ADRA1B and ADRA2A expressed by HTPCs, cultured with or without FCS (extracted from the data of the study by [17]); Figure S2: Dose response experiment with cultured HTPCs (*n* = 1) to increasing concentrations of clonidine ranging from 1 µM, 10 µM, 100 µM and 1000 µM over a 24 h period. Images of cells 10 min, 3 h and 24 h after treatment as well as PBS control are depicted; Figure S3: Diagram of gel contraction assay with HTPCs. The percentage of the gel contraction was measured after 3 h, 24 h and 24 h + 1 h FCS treatment under control conditions, clonidine treatment and FCS treatment (positive control). Clonidine treatment results in reduced (not significant) contraction of the cells in contrast to control conditions. Bars show mean with SD (*n* = 3); Figure S4: Mass spectrometry analysis of HTPCs with and without 24 h treatment with 10 µM clonidine revealed no major alterations in their proteome profiles. HTPCs were derived from 5 different donors. (A) Volcano plot analysis of protein intensity values of clomidine-treated (10 µM, 24 h) and untreated controls. Statistical evaluation was carried out using the Welch’s *t*-test. The permutation-based FDR significance cutoff (*p* < 0.05) is depicted by the black curves. (B) Heatmap and unsupervised hierarchical clustering of label-free quantification intensity values for untreated (control = CO) and clonidine-treated (clonidine = CLO; 10 µM, 24 h) treated HTPCs. (C). Multi scatter plots and Pearson correlation analysis demonstrates high homogeneity of biological replicates.
