# Supplementary material for: Identification of Catecholamine and Drug Target α2A-Adrenoceptor in Human Testis and Human Testicular Peritubular Cells
Source: J Clin Med. 2024 Jul 25;13(15):4357. doi: 10.3390/jcm13154357 (PMC11313226; doi:10.3390/jcm13154357)
Supplement: Supplementary file 1 [file jcm-13-04357-s001.zip › jcm-3072648-supplementary.pdf]

Journal of
Clinical Medicine

MDPI

Supplementary Materials

Table S1. Information of PCR-Primer (AT: Annealing Temperature).

Primer Name	Sequence (5´- 3´)	PCR-Product (bp)	AT (° C)	Accession Number
GAPDH	*For* GAGCGAGATCCCTCCAAAAT *Rev* AAATGAGCCCCAGCCTTCT	101	60	NM_002046.7
COX2	*For* CTTACCCACTTCAAGGGA *Rev* GCCATAGTCAGCATTGTAAG	132	57	NM_000963.4
IL6	*For* AACCTGAACCTTCCAAAGATGG *Rev* TCTGGCTTGTTCCTCACTACT	159	62	NM_000600.4
IL8	*For* TCTTGGCAGCCTTCCTGA *Rev* GAATTCTCAGCCCTCTTC	271	60	NM_000584.4
ADRA2A	*For* AGAAGTGGTACGTCATCTCGT *Rev* CGCTTGGCGATCTGGTAGA	97	59	NM_000681.4
MCP1	*For* AGGTGACTGGGGCATTGAT *Rev* GCCTCCAGCATGAAAGTCTC	109	58	NM_002982.4
StAR	*For* ACGTGGATTAACCAGGTTCG *Rev* CAGCCCTCTTGGTTGCTAAG	149	58	NM_000349
CXCL12	*For* TCAGCCTGAGCTACAGATGC *Rev* CTTTAGCTTCGGGTCAATGC	161	60	NM_199168.4
DCN	*For* GGAATTGAAAATGGGGCTTT *Rev* GCCATTGTCAACAGCAGAGA	221	59	NM_001920.5
BGN	*For* TGGCCTGAAGCTCAACTACC *Rev* GCCTGGATTTTGTTGTGGTC	111	59	NM_001711.6
Col I	*For* CACACGTCTCGGTCATGGTA *Rev* AAGAGGAAGGCCAAGTCGAG	91	58	NM_000088.4

AR	*For* AGCCTCAATGAACTGGGAGA *Rev* TCCTGGAGTTGACATTGGTG	175	62	NM_000044.6

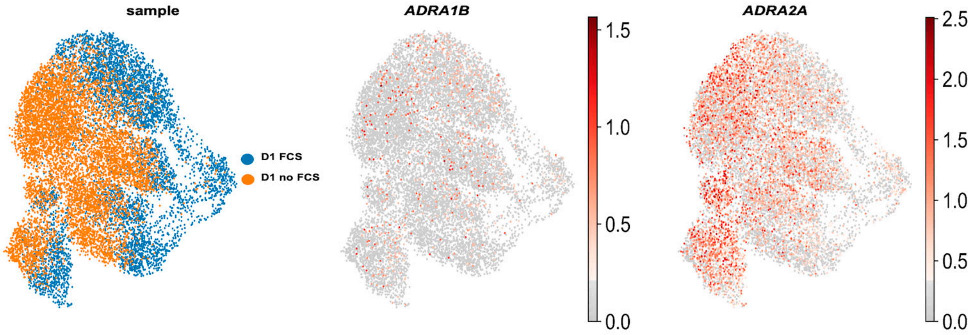

Figure S1.

UMAP plot of *ADRA1B* and *ADRA2A* expressed by HTPCs, cultured with or without FCS (extracted from the data of the study by Liebich et al., 2022).

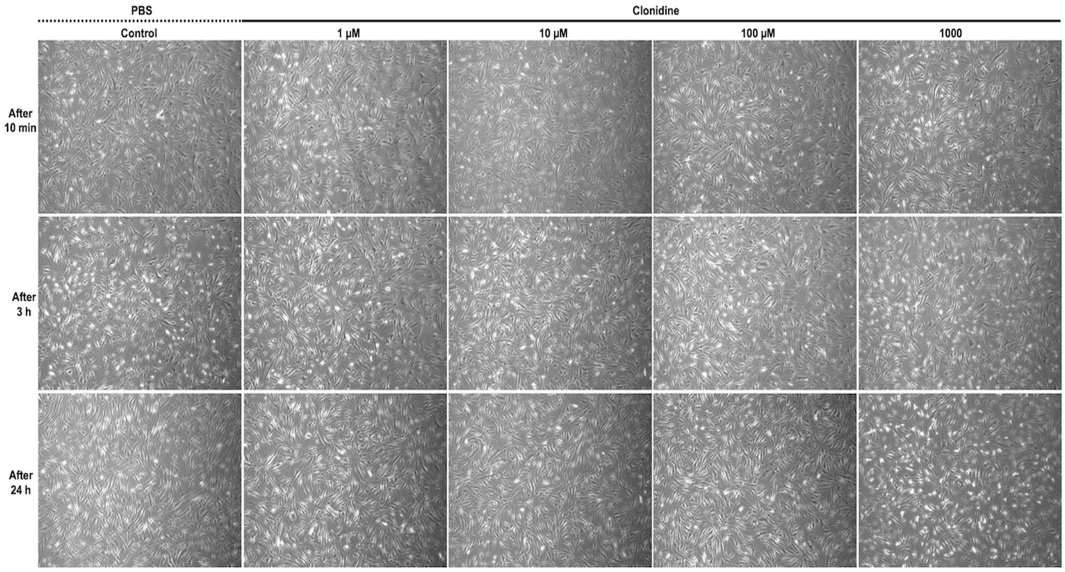

Figure S2.

Dose response experiment with cultured HTPCs (n = 1) to increasing concentrations of clonidine ranging from 1 µM, 10 µM, 100 µM and 1000 µM over a 24 h period. Images of cells 10 min, 3 h and 24 h after treatment as well as PBS control are depicted.

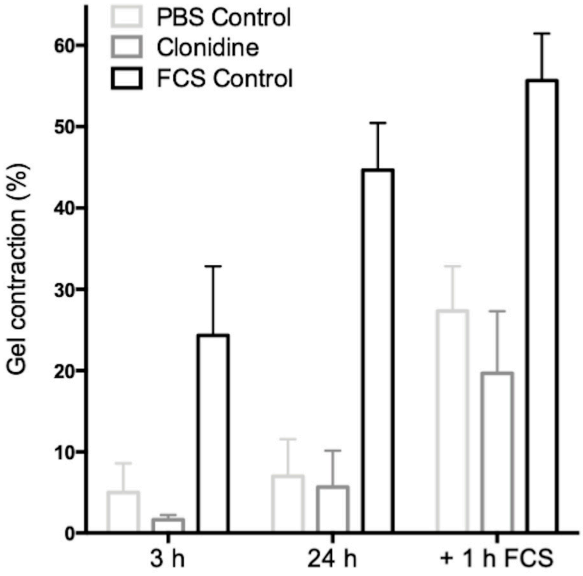

Figure S3.

Diagram of gel contraction assay with HTPCs. The percentage of the gel contraction was measured after 3 h, 24 h and 24 h + 1 h FCS treatment under control conditions, clonidine treatment and FCS treatment (positive control). Clonidine treatment results in reduced (not significant) contraction of the cells in contrast to control conditions. Bars show mean with SD (n = 3).

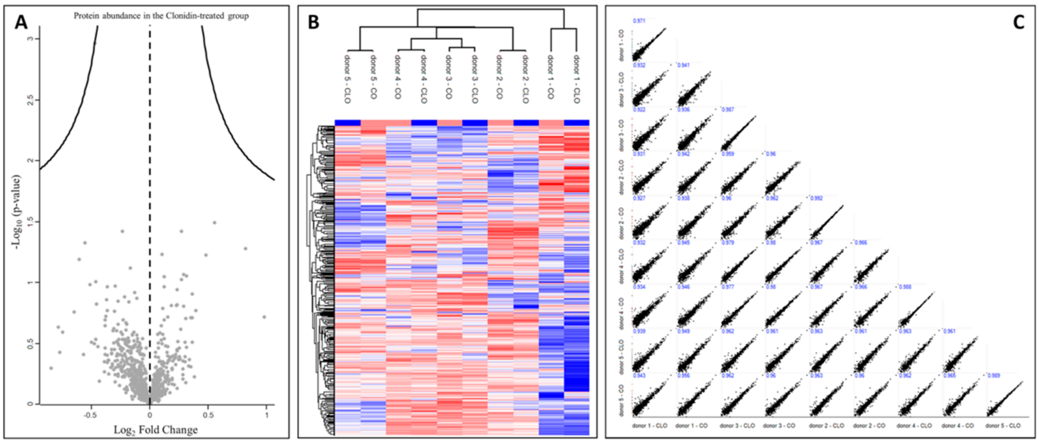

Figure S4.

Mass spectrometry analysis of HTPCs with and without 24 h treatment with 10 µM clonidine revealed no major alterations in their proteome profiles. HTPCs were derived from 5 different donors.

(A) Volcano plot analysis of protein intensity values of clomidine-treated (10 µM, 24 h) and untreated controls. Statistical evaluation was carried out using the Welch's t-test. The permutation-based FDR significance cutoff ($P < 0.05$) is depicted by the black curves.

(B) Heatmap and unsupervised hierarchical clustering of label-free quantification intensity values for untreated (control = CO) and clonidine-treated (clonidine = CLO; 10 µM, 24 h) treated HTPCs.

(C). Multi scatter plots and Pearson correlation analysis demonstrates high homogeneity of biological replicates.
